# Quality Evaluation of Pharmaceutical Formulations Containing Hydrochlorothiazide

**DOI:** 10.3390/molecules191016824

**Published:** 2014-10-20

**Authors:** Marcelo Antonio de Oliveira, Maria Irene Yoshida, Daphne Carina Gonçalves Monteiro da Silva

**Affiliations:** 1 Departamento de Ciências da Saúde, Centro Universitário Norte do Espírito Santo, UFES, Rodovia BR 101 Norte, km 60, 29932-540 São Mateus, ES, Brazil; 2 Departamento de Química, Universidade Federal de Minas Gerais, Av. Pres. Antônio Carlos, 6627-31270-901 Belo Horizonte, MG, Brazil

**Keywords:** hydrochlorothiazide, quality control, dissolution profile, polymorphism

## Abstract

Hydrochlorothiazide is a diuretic used to treat hypertension that belongs to class IV of the Biopharmaceutics Classification System. The drug was evaluated by quality control, thermal characterization tests, and pharmaceutical formulation compatibility studies. It was concluded that the generic drug, Lab 2, was not a pharmaceutical equivalent. The compounded drugs, Lab 5 and Lab 6, produced unsatisfactory but expected results, since there is no requirement for dissolution and dissolution profile testing for the commercialization of these products. In a compatibility study, lactose and mannitol were shown to be incompatible with HCTZ, which may explain the lack of equivalence of the generic pharmaceutical product, associated with other situations.

## 1. Introduction

Hydrochlorothiazide (HCTZ) is a diuretic of the thiazide class, which is used in the treatment of edema, hypertension, congestive heart failure and different forms of renal and hepatic dysfunction [1]. 

This drug has the molecular formula C_7_H_8_ClN_3_O_4_S_2_ and its molecular weight is 297.74 g·mol^−1^. It is presented in the form of a white or almost white crystalline powder, which is odorless and has a melting point in the range 266.0 to 270.0 °C. HCTZ is soluble in acetone and dilute alkaline solutions [2].

In accordance with the Biopharmaceutics Classification System, HCTZ is classified as being of low solubility and low permeability, belonging to Class IV [3]. These characteristics limit its therapeutic action, as these drugs are prone to exhibit scant oral bioavailability [4].

HCTZ is found in drugstores in the form of tablets as a generic or similar drug, and pharmaceutical capsules produced by compounding pharmacies. The reference product is Clorana^®^, indicated by the National Agency of Sanitary Vigilance (ANVISA) which is also marketed [5,6]. It should be noted that the Brazilian Society of Cardiology suggests the use of industrially produced medicines for the chronic treatment of hypertension, mainly due to variability in the quality of compounded medications.

For the manufacture of compounded drugs, the pharmacy must follow the norms related to Resolution-RDC 67, which describes good manufacturing practices for compounded drugs, and presents norms for the manufacture of products with high quality [7]; also, the Brazilian Pharmacopoeia defines the product quality tests and acceptance criteria [2]. Moreover, the industrially produced medicines, either generic or similar medicines, must comply with Resolution RDC 31 [8] which provides for the studies of pharmaceutical equivalence and comparative dissolution profiles, which are tests that are used to ensure the pharmaceutical equivalence of similar drugs and generic drugs [9,10]. It is noted that Brazilian laws cited for the production of compounded, similar or generic drugs have international quality standards, because ANVISA follows the U.S. Food and Drug Administration (FDA) and International Conference on Harmonization (ICH) protocols.

Issues related crystal polymorphism may still be present in HCTZ. Polymorphism is defined as the presence of the same substance in different crystalline forms, and these crystalline forms may have different physicochemical properties. Polymorphism may also include solvated or hydrated (pseudopolymorphism) products and amorphous forms. This phenomenon may lead to changes in pharmaceutical products with regard to their purity parameters, stability, quality or efficiency, and the bioavailability of pharmaceutical product, which may lead to alterations in the* in vivo* effect [11]. The guide published by the FDA establishes criteria to characterize the polymorphic form and the degree of criticality in the final product [12]. The techniques listed in this characterization are: X-ray diffraction, thermal analysis, microscopy and spectroscopy. For generic drugs, the ICH Guidance (2007) sets criteria about the importance of polymorphism, its characterization, its influence on the quality of the pharmaceutical product, and the degree of criticality of the polymorphism in the product [13].

The study of expiration dates is referred to as a stability study. This is one of the main factors evaluated in the development of pharmaceutical formulations. These studies are routinely conducted by the pharmaceutical industry; however, it requires extended periods of sample storage under controlled temperature and humidity conditions [14,15]. Although these do not replace conventional studies, thermoanalytical techniques such as DSC have been shown to be very helpful in stability studies, allowing the selection of stable formulations with extreme rapidity through compatibility studies [16,17,18,19,20].

In the evaluation of compatibility by DSC, an interaction can be viewed as a change in melting point, a change in peak area, the appearance of a transition, or the appearance and disappearance of peaks after mixing the components. However, after the binary mixture of components, there is invariably some change in the transition temperature of the shape, and in the area of the peaks; this cannot be a damaging interaction and should be interpreted with caution. If the excipient is reactive and incompatible with the drug, this must be avoided. When there is suspicion of a chemical reaction and/or interaction, but the thermal changes are small, the incompatibility should be confirmed by other analytical techniques such as HPLC. The advantage of DSC in relation to the traditional technique of compatibility studies of pharmaceutical formulation is that it is not necessary to store the samples for long periods before evaluation. These samples could be stored under extreme conditions of temperature and humidity to accelerate interactions, as recommended by Brazilian law [14].

Therefore, it is essential to compare the quality of compounded drugs in relation to industrially produced medicines, mainly evidencing the dissolution profiles of these drugs, regardless of whether they are reference, generic, similar or compounded drugs.The aim of this paper was to evaluate capsules containing 50 mg of HCTZ obtained from compounding pharmacies, and compare these with industrially produced 50 mg HCTZ medicines in tablet form. In addition, the compatibility of the pharmaceutical formulations described by the manufacturers was evaluated by DSC in order to indicate possible incompatibility and correlate these results with those of dissolution of the drug in the dissolution profile tests.

## 2. Results and Discussion

### 2.1. Quality Control Tests

The results of the quality control tests, performed as recommended by the Brazilian Pharmacopoeia [2], as shown in Table 1, indicate that only the Lab 2, Lab 5 and Lab 6 products would be rejected, according to their low dissolution value. The dissolution of each drug was studied using the F2 and DE, for better understanding. 

**Figure 1:**
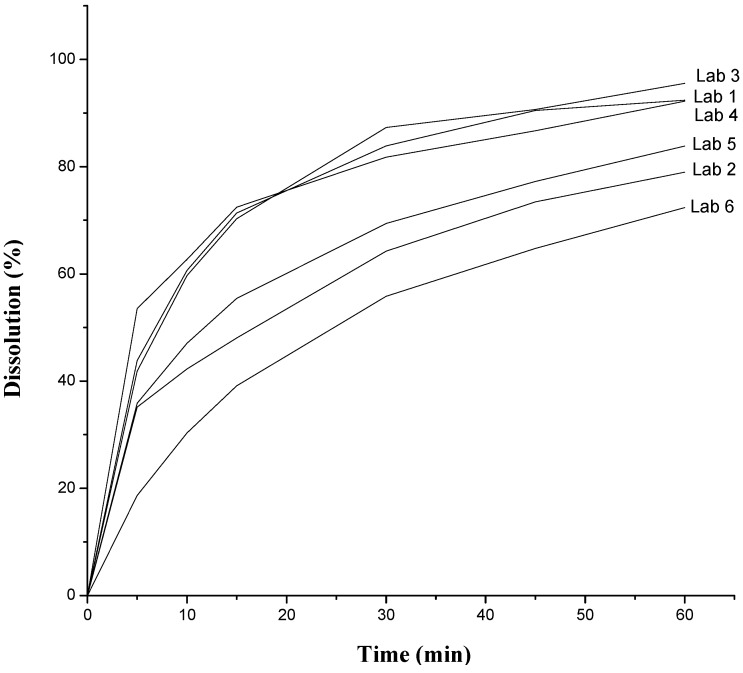
Dissolution profile of the six laboratory products evaluated.

**Figure 2:**
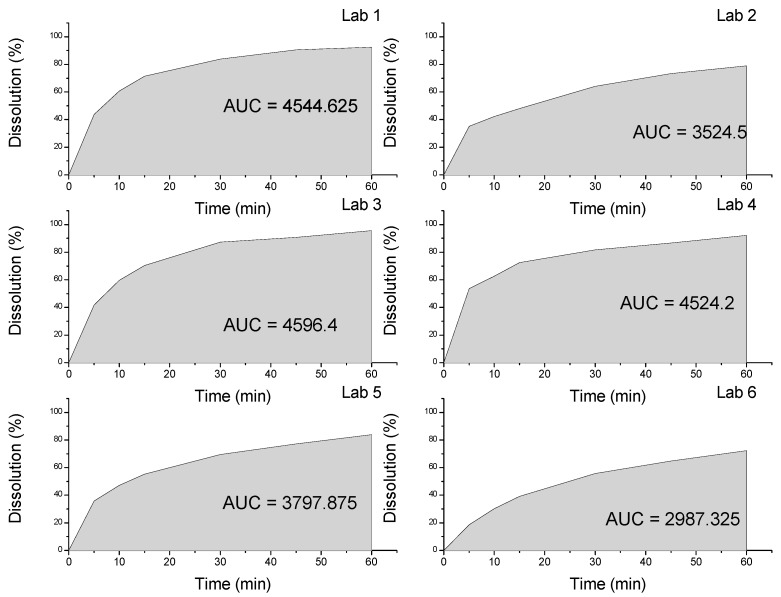
Dissolution profile of the six laboratories evaluated for determination of the AUC.

Figure 1 shows the dissolution profiles for the six labs evaluated. The results demonstrate that the dissolution profile for Lab 2, 5, and 6 did not reach the minimum acceptable dissolution. In addition, the calculated values of F2, which were 19, 26 and 9, respectively, for the three laboratories were unsatisfactory as these drugs were considered pharmaceutical equivalents when compared to the reference product (Lab 1). For Labs 3 and 4, the F2 value was acceptable, as these were 91.3 and 76.7, respectively. The dissolution efficiency (DE, %) was calculated for all samples, using the AUC, as described in Figure 2, and considering that the equivalent area to 100% dissolution on time of 60 min was 6000. Thus, the DE was as follows: Lab 1 = 75.74%; Lab 2 = 58.74%; Lab 3 = 76.60%; Lab 4 = 75.40%; Lab 5 = 63.30%, and Lab 6 = 49.78%. The results also showed large differences between the medicines from Labs 2, 5 and 6 with respect to the reference drug (Lab 1).

By analyzing the excipients contained in all of the studied drugs, and considering that the drug with the appropriate dissolution that serves as a parameter for the development of new products is the reference drug (Lab 1), some observations can be made. The two formulations that achieved an appropriate release, Lab 3 and Lab 4, were very different. Lab 3 shows the super-disintegrant sodium starch glycolate, which is associated with the soluble diluent lactose, which may be responsible for the adequate dissolution. The obtained pharmaceutical equivalence to Lab 4 may be associated with the combination of starch and povidone disintegrants, which are associated with the soluble diluent mannitol.

**Table 1:** Results of quality control of medicines Lab 1, Lab 2, Lab 3, Lab 4, Lab 5 and Lab 6.

Test	Specifications	Lab 1 (Ref)	Lab 2 (Gen)	Lab 3 (Sim)	Lab 4 (Sim)	Lab 5 (Comp)	Lab 6 (Comp)
Identification	Identification by TLC	Approved	Approved	Approved	Approved	Approved	Approved
Hardness	Minimum 30 N	>32 N	>45 N	>55 N	>30 N	not applicable	not applicable
Disintegration	Disintegrates in water at 37 °C within 30 min	<1 min	<1 min	<1 min	<1 min	<1 min	<1 min
Weight	−7.5% < X < +7.5%	Approved	Approved	Approved	Approved	Approved	Reproved
Friability	Maximum 1.5%	0.04%	0.06%	0.006%	0.11%	not applicable	not applicable
Uniformity of dosage units	85.0 < X < 115.0% VR, RSD < 6.0%	Average = 220.75% RSD = 1.12%	Average = 243.76% RSD = 2.76%	Average = 203.85% RSD = 1.1%	Average = 172.75% RSD = 1.35%	Average = 183.60% RSD = 3.13%	Average = 198.85% RSD = 3.7%
Dissolution	60% (Q) in 30 min	83.89%	64.22%	87.28%	81.77%	33.27%	55.83%
Assay	Minimum 93.0%LV and maximum 107.0%LV	94.88%	98.43%	100.57%	99.72%	101.28%	97.87%

Where: LV = Labeled Value; Ref = reference drug; Gen = generic drug; Sim = similar drug; Comp = compounded drug.

Although Lab 4 did not present a super-disintegrant in the formulation, and as HCTZ is a drug of low solubility, the solubilization/dissolution of the drug was adequate compared to the reference drug (Lab 1). The results for Lab 3 and Lab 4 were expected because the dissolution profile test is required for the registration of these drugs as similar [9,10]. 

For Lab 2, labeled as a generic drug, the formulation described indicates that the drug would be readily solubilized. Lab 2 shows three super-disintegrants, crospovidone, croscarmellose sodium and sodium starch glycolate, associated with the surfactant sodium lauryl sulfate, and even presents a soluble diluent such as lactose; these are all excipients that promote disintegration and dissolution. However, the dissolution was not appropriate and this may be related to some incompatibilities in the pharmaceutical formulations, or inadequate storage conditions of the drug in question which may have led to failure of the same, or even a lack of quality between lots of the generic product.

Lab 5 and Lab 6 are compounded drugs in the form of capsules, and presented inadequate dissolution profiles. The two formulations presented by manufacturers use starch as a diluent, which is an insoluble diluent often used as a disintegrant in tablets. In the capsule formulations, the most commonly used diluent in the market is lactose, which is a soluble diluent and helps with the solubilization of drugs; however, this is not used by Lab 5 and Lab 6, even though the low solubility of HCTZ is known. Although Lab 5 used sodium lauryl sulfate as an excipient to help with solubilization, the results were not satisfactory.

### 2.2. Thermal Characterization and Compatibility Study of Pharmaceutical Formulation 

**Figure 3:**
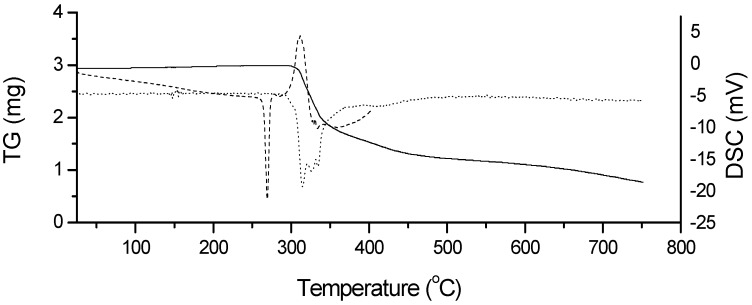
Dynamic TG (^_____^), DTG (^......^) and DSC (----) curves for HCTZ, under nitrogen atmosphere and a heating rate of 10 °C·min^−1^.

HCTZ showed thermal stability up to 293 °C, as observed in a dynamic TG curve (Figure 3). This showed the onset of melting at 265.0 °C, a melting peak at 269.2 °C with a characteristic endotherm and a melting heat of 316.42 J·g^−1^, as shown in the DSC curve in Figure 3. After the fusion, there was an exothermic event with a peak at 311.6 °C. This exothermic event coincided with the thermal degradation of 74.86% in three steps, as presented in the dynamic TG curve of the drug, and evidenced by DTG (derivative thermogravimetry).

**Figure 4:**
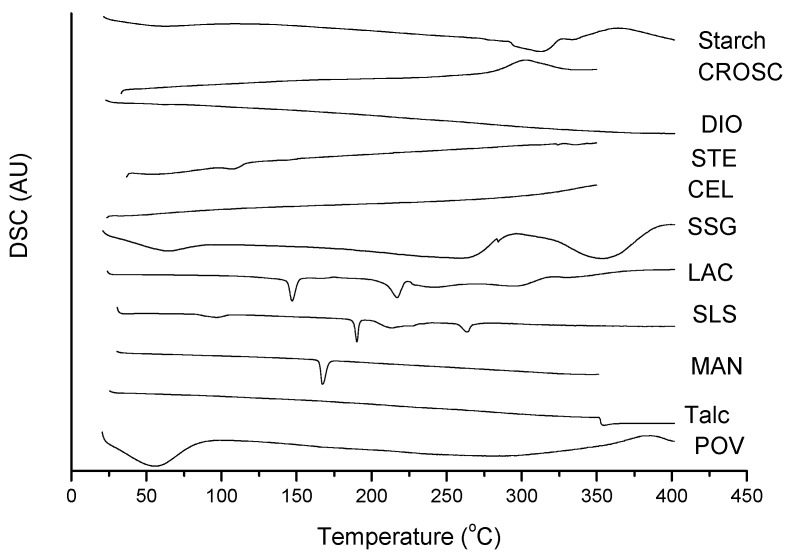
DSC curves of excipients of HCTZ tablets, under nitrogen atmosphere and a heating rate of 10 °C·min^−1^.

Figure 4 shows the DSC curves of the excipients used in the formulations of HCTZ: starch, sodium starch glycolate (SSG), lactose (LAC), sodium lauryl sulfate (SLS), povidone K-30 (POV), talc, colloidal silicon dioxide (DIO), magnesium stearate (STE), croscarmellose sodium (CROSC), microcrystalline cellulose (CEL) and mannitol (MAN). Under the analysis conditions, three excipients showed fusion below 265 °C, which is the melting temperature of HCTZ; these are lactose, mannitol and sodium lauryl sulfate, which melted at 147.1 °C, 190.1 °C and 167.4 °C, respectively.

**Figure 5:**
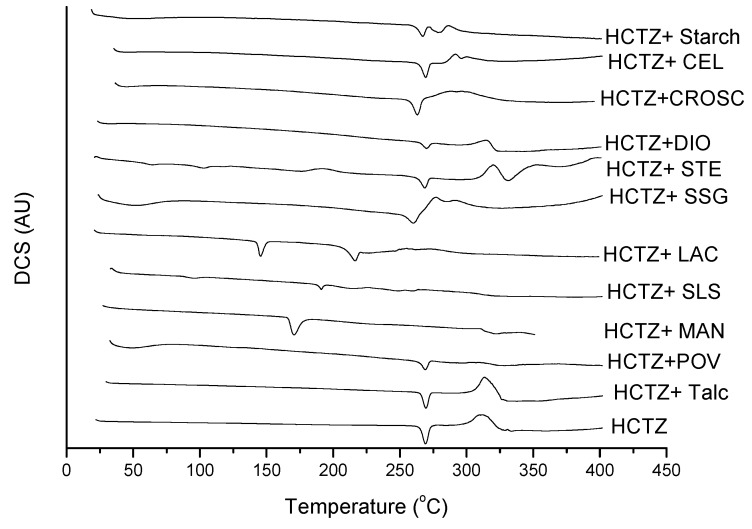
DSC curves of binary mixtures of HCTZ with each excipient (1:1), under nitrogen atmosphere and a heating rate of 10 °C·min^−1^.

Figure 5 shows the DSC curves for the binary mixtures of each drug with each excipient, in 1:1 ratios. The disappearance of the melting peak of HCTZ can be observed after combination with lactose, sodium lauryl sulfate and mannitol. This disappearance may be associated with a chemical incompatibility or may simply be associated with the solubilization of HCTZ in the molten mass of the excipient [16,17,19].

**Figure 6:**
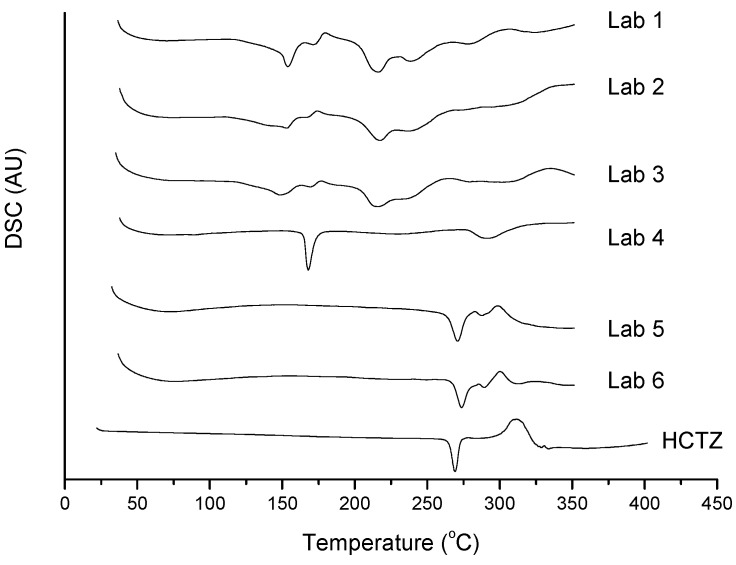
DSC curves of multicomponent mixtures (manufacturer’s laboratories) for HCTZ, under nitrogen atmosphere and a heating rate of 10 °C·min^−1^.

Figure 6 shows the DSC curves for multi-component mixtures (medicines) and the absence of the melting peak of HCTZ can be observed for formulations Lab 1 to Lab 4, which coincidentally have the excipients lactose, mannitol and sodium lauryl sulfate in their formulations.

**Figure 7:**
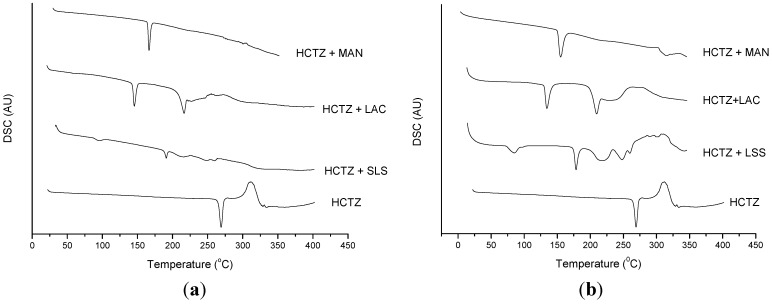
DSC curves of binary mixtures of HCTZ with each excipient (1:1), which presented the possibility of incompatibility, under nitrogen atmosphere and heating rates of (**a**) 10 °C·min^−1^ and (**b**) 25 °C·min^−1^.

One technique used to discard the possibility of solubilization of the drug (HCTZ) in the mass of excipient which was already merged is to increase the heating rate of the DSC, usually up to 25 °C·min^−1^ [16]. Thus, the drug would not be fused completely, and a small amount of solid would be sufficient to show the melting peak of HCTZ by DSC. Figure 7 shows the DSC curves for binary mixtures of HCTZ with the possible incompatible excipients at two heating rates: 10 °C·min^−1^ and 25 °C·min^−1^. In the presence of sodium lauryl sulfate at 25 °C·min^−1^, it is possible to observe the HCTZ peak. However, when HCTZ is associated with lactose or mannitol, this does not occur, and there is no evidence of the melting peak of HCTZ at higher heating rates, characterizing chemical incompatibility. The chemical interaction between amines and lactose is already known and has been cited by some authors [21,22]; this is derived from the Maillard reaction, which can be viewed by DSC.

In multicomponent mixtures, the DSC curves at a heating rate of 25 °C·min^−1^ showed the same behavior as the curves at 10 °C·min^−1^. For Labs 1 to 4, the HCTZ melting peak disappeared in the presence of lactose and mannitol in the four formulations, while in formulations 5 and 6, the fusion of the drug became evident.

## 3. Experimental

### 3.1. Origin of Samples 

The drugs evaluated were acquired in the Brazilian market. Table 2 identifies the laboratory (Lab), the pharmaceutical formulation and the type of drug.

**Table 2:** Laboratories, pharmaceutical formulation and drug type.

Laboratory	Pharmaceutical Formulation	Type Drug
Lab 1	HCTZ, lactose monohydrate, starch, and magnesium stearate.	Reference
Lab 2	HCTZ, sodium lauryl sulfate, croscarmellose sodium, crospovidone, sodium starch glycolate, lactose, microcrystalline cellulose, magnesium stearate, colloidal silicon dioxide.	Generic
Lab 3	HCTZ, microcrystalline cellulose, colloidal silicon dioxide, lactose, magnesium stearate, sodium starch glycolate.	Similar
Lab 4	HCTZ, starch, magnesium stearate, mannitol, talc, povidone (PVP) and ethanol.	Similar
Lab 5	HCTZ, magnesium stearate, Aerosil^®^ (colloidal silicon dioxide), talc, corn starch, sodium lauryl sulfate.	Compounded
Lab 6	HCTZ, Aerosil^®^ (colloidal silicon dioxide), microcrystalline cellulose and starch.	Compounded

### 3.2. Quality Control Tests

Tests for Quality Control were performed according to methods recommended by the Brazilian Pharmacopoeia [2]. The procedures used for the assay, dissolution and dissolution profile were as follows.

#### 3.2.1. Assay

The samples and standard solutions were diluted with 0.1 M NaOH to a final concentration of 0.015 mg·mL^−1^. The readings were taken in the UV spectrum at 273 nm using 0.1 M NaOH as a blank.

#### 3.2.2. Dissolution and Dissolution Profile

The dissolution profile was performed as recommended in the Brazilian Pharmacopoeia [2] for dissolution, considering the collection times of 5, 10, 15, 30, 45 and 60 min after the initiation of dissolution. The conditions used were:

- Dissolution medium: 0.1 M hydrochloric acid, 900 mL.
- Apparatus 1 (basket): 100 rpm.
- Collection times in the dissolution profile: 5, 10, 15, 30, 45 and 60 min.
- Procedure: at the specified times, 15 mL aliquots of dissolution medium were taken, and filtered through filter paper. Then, 10 mL of the filtrate was pipetted into a 50 mL volumetric flask, and made up to the final volume with 0.1 M hydrochloric acid. The absorbance was measured at 272 nm using the same solvent for zero adjustment. The amount of C_7_H_8_ClN_3_O_4_S_2_ dissolved in the medium was calculated by comparing the readings obtained with the standard solution of hydrochlorothiazide at 0.01 mg·mL^−1^ prepared in the same solvent.
- Tolerance: No less than 60% (Q) of the labeled amount of C_7_H_8_ClN_3_O_4_S_2_ should dissolve within 30 min.

The dissolution profile is a comparative study with analytical tests and multiple collection times, which allows evaluation of the dissolution of a particular drug and the comparison of two formulations. Graphs were plotted and comparison of the dissolution profiles of different formulations was carried out by calculating the similarity factor (F2), according to Equation (1). According to RDC 31/2010, for two dissolution profiles to be considered similar, the similarity factor (F2) must be between 50 and 100. The collection times should be the same for both formulations and the number of collection points must be representative (until a plateau in the curve is obtained); there must also be at least five time points, for use in the calculation of F2. The coefficients of variation for the first sampling points should not exceed 20% and the remaining points must be a maximum of 10% [8]:

(1) $$F2=50x\log\left\{{\left[1+\left(\frac{1}{n}\right)\sum_{t=1}^{n}{\left(Rt-Tt\right)}^{2}\right]}^{-0,5}x100\right\}$$

where: *n* = number of collection times; Rt = percentage value dissolved at time t, obtained with the reference product; and Tt = percentage value dissolved at time t of the test product.

Another way of comparing the dissolution profile is calculated by determining the dissolution efficiency (DE), which was introduced in 1972 by Khan and Rhodes, being determined by the ratio in percentage of the area under curve (AUC) obtained from the profile dissolution, and the total area of the rectangle considered as 100% dissolution for the same time interval. The DE was calculated using Equation (2) [23]:

(2) $$ED=\frac{\overset{t}{\underset{0}{\text{∫}}}yxdt}{y100xt}x100$$

where $\overset{t}{\underset{0}{\text{∫}}}yxdtx100$ = area under curve in an interval of time t, expressed in percentage value; and y100xt = rectangle area considering 100% dissolution in the same time t.

The results obtained from the F2 (similarity factor) and DE (dissolution efficiency) can be, theoretically, correlated with the* in vivo* data, since the bioavailability can be determined by the area under the absorption curve for a particular drug as a function of time [8,9,10,23,24].

### 3.3. Thermal Characterization of Drugs and Compatibility Study of Pharmaceutical Formulation

Thermal characterization of the drug was performed by thermogravimetric analysis (TG) and Differential Scanning Calorimetry (DSC). TG curves were obtained on a Shimadzu thermobalance DTG60 with a heating rate of 10 °C·min^−1^, heating to 750 °C, under nitrogen atmosphere with a flow rate of 50 mL·min^−1^, using an alumina crucible and with a sample mass about 5.0 mg. The DSC curves were obtained under the same conditions described for TG.

To evaluate the compatibility of the studied pharmaceutical formulations, DSC trials were conducted with samples of: (a) HCTZ; (b) excipients present in the formulations of medicines: reference, generic, similar and compounded drugs; (c) 1:1 binary mixtures of excipient and HCTZ, with the objective of increasing the probability of drug/excipient interactions; and (d) commercialized drugs (multicomponent mixtures).

The DSC curves were obtained on a Shimadzu DSC50 cell under a nitrogen atmosphere, with a flow rate of 50 mL·min^−1^, and a heating rate of 10 °C per minute up to a temperature of 400 °C. The samples were weighed on an analytical balance; all had a mass of around 0.8 mg. Samples were placed in a partially closed aluminum crucible, and were then analyzed by DSC.

## 4. Conclusions

The generic drug, Lab 2, was found to not be a pharmaceutical equivalent; the cause for this may be associated with the presence of lactose, which has been shown to be incompatible with HCTZ; there may have also been a lack of quality between lots for generic drugs; or inadequate storage conditions of the drug in question which may have led to failure of the same.

Similar drugs, Lab 3 and Lab 4, were pharmaceutical equivalents with respect to the reference product (Lab 1), which was already expected due to the quality requirements for their registration and production.

For compounded drugs, Lab 5 and Lab 6, the unsatisfactory dissolution results and the dissolution profiles were expected because there is no requirement for these tests during marketing. HCTZ showed thermal stability up to 293 °C, the melting peak was at 269.2 °C and there was a characteristic melting heat endotherm of 316.42 J·g^−1^. After the merger, the degradation was found to occur in three stages, with the exothermic peak occurring at 311.6 °C.

In a compatibility study, conducted using DSC, lactose and mannitol were shown to be incompatible with HCTZ. In summary, this work demonstrates the importance of the use of medicines manufactured in pharmaceutical industries, where there are greater requirements about the quality of products. The compounded drugs in pharmacies have lower quality. Note also the importance of avoiding pharmaceutical formulations of HCTZ containing mannitol and lactose, which are incompatible with the drug and may change the quality of the pharmaceutical product.
